# First results with the immediate reconstructive strategy for internal hardware exposure in non-united fractures of the distal third of the leg: case series and literature review

**DOI:** 10.1186/1749-799X-7-30

**Published:** 2012-08-28

**Authors:** Luca Vaienti, Adriano Di Matteo, Riccardo Gazzola, Luca Pierannunzii, Giovanni Palitta, Andrea Marchesi

**Affiliations:** 1 Plastic Surgery Department, Università degli Studi di Milano, IRCCS Policlinico San Donato, Piazza Malan, 20097 San Donato Milanese, Milan, Italy; 2 Istituto Ortopedico Gaetano Pini, P.zza C. Ferrari, 1, Milan, Italy

**Keywords:** Exposure, Flap, Internal fixator, Reconstruction, Leg, Fracture, Internal hardware exposure, Infection, Immediate reconstruction, Pedicled flaps, Lower limb

## Abstract

**Background:**

Fractures of the distal third of the leg are increasingly common and are often handled by open reduction and internal fixation. Exposure and infection of internal hardware could occur, especially after high energy traumas, requiring hardware removal and delayed soft tissue reconstruction. Nevertheless immediate soft tissue reconstruction without internal hardware removal is still possible in selected patients.

In this study the effectiveness and the complications of immediate soft tissue reconstruction without internal hardware removal is analyzed.

**Methods:**

13 patients, affected by internal hardware exposure in the distal leg, treated with immediate soft tissue reconstruction with pedicled flaps and hardware retention, are retrospectively analyzed, with special regard to flap survival and wound infection.

**Results:**

Wound infection was observed in 10 cases before surgery and in 5 cases surgical debridement was necessary before reconstruction which was performed in a separate operative session.

After reconstruction, wound dehiscence and infection occurred in 5 cases, and in 3 cases removal of internal hardware was necessary in order to achieve the complete healing of dehiscence. In one case the previous flap failed but prompt reconstruction with a sural fasciocutaneous flap was performed without hardware removal and without complications. Pre-operative infection and late reconstructive surgery are predictive for higher rates of post-operative complications (respectively p 0.018 and p 0.028).

**Conclusion:**

Our approach achieved full recovery in 53.8% of the treated cases after one-step surgery, therefore reducing hospitalization and allowing early mobilization. Controlled trials are needed to confirm the effectiveness of this strategy, although the present case series shows encouraging results.

## Introduction

Hardware exposure in the lower limb represents a demanding problem for the Reconstructive Surgeon who has to consider both the tegumental status and the underlying orthopedic features.

Fractures of the distal third of leg, like tibial metaphyseal fractures, malleolar fractures and pilon fractures, are increasingly common, often caused by low energy traumas (due to the aging of population and increased activity of the elderly), but sometimes provoked by high-energy trauma in young and active patients [1]. This kind of injuries is usually treated with Open Reduction Internal “rigid” Fixation (ORIF) which is quite safe and effective in low-energy fractures [2-5], while for high energy plafond injuries, external fixation is often recommended in order to reduce the soft tissue damage [6,7].

All the malleolar fractures and the majority of distal tibia fractures are commonly approached with ORIF, as confirmed by McFerran’s study [8] in which 89% of fractures were treated by open reduction and internal fixation. Among the pilon fractures treated with ORIF, 50% to 54% of complications have been reported, including wound dehiscence and infections [7,8]. The complication rate reaches 70% in Ruedi type III fractures [9-12].

Internal hardware exposure on the distal third of leg is a challenging problem due the thin cutaneous layer which is supplied by a poor and fragile vascular network. The importance of a prompt soft tissue reconstruction has been emphasized in literature [13]. In fact this complication may lead not only to wound breakdown and deep infection, but also to algodystrophy, delayed fracture healing, joint stiffness and poor functional outcome [14-16].

When internal hardware exposure occurs, literature suggests serial irrigations, debridement, antibiotic therapy and hardware removal [17]. Soft tissues reconstruction is traditionally performed after removal of the hardware, nevertheless, whenever hardware removal may destabilize the fracture, external fixation could be evaluated before tegumental reconstruction.

Nieminen [13] describes 15 cases of internal hardware exposure in tibial fractures, treated by microvascular flaps after hardware removal. In all his cases the internal hardware was removed before tegumental reconstruction and in eight patients the fracture was stabilized with external fixation. Full recovery after one-step surgery was reported in 9 patients. In the remaining patients, one to five additional surgeries were necessary and in one case and a below-knee amputation was required.

Although soft-tissue reconstruction after early removal of internal hardware constitutes the preferred treatment, hardware salvage with pedicled local or free flaps on the exposed hardware is still possible. According to Mathes, soft tissue coverage of exposed hardware and eventually osteomyelitis is an effective strategy [18].

In line with this theory, Tan et al [19] that analyzed the results of pedicled muscle flaps applied after the eradication of the infection on exposed internal hardware in nine patients. Salvage of internal hardware was achieved in 4 cases.

Given the importance of timing in free tissue transfer reconstruction underlined by Nieminen [13], the main goal for exposed internal hardware in the lower limb should consist in the prompt treatment by wound debridement and flap coverage, without additional orthopedic surgeries.

Literature is lacking about the chance of immediate coverage of exposed internal hardware, underlying the necessity of further studies that deepen timings, complication rates and indications of immediate soft tissue reconstruction.

In this study we aim to compensate for these issues analyzing retrospectively a series of homogeneous cases managed with a standard reconstructive protocol, called Immediate Reconstructive Strategy (IRS).

## Materials and methods

In this study we retrospectively analyzed 13 patients treated for internal hardware exposure in distal leg fractures, from July 2004 to march 2010.

All of them were managed with a common protocol based on soft tissue reconstruction with local pedicled and free flaps and hardware retention (IRS, Immediate Reconstructive Strategy).

Each patient suffering from hardware exposure after ORIF in the distal third of the leg, was indicated for IRS at our institution. All the fractures that looked healed on standard radiograms were excluded, as hardware removal was considered safer in such cases.

### Operative procedure

On admission a written informed consent was obtained from the patient for publication of this report and any accompanying images. A microbiological culture swab of the wound was then obtained and C Reactive Protein (CRP) was tested.

Intramedullary, localized or diffuse osteomyelitis was excluded with blood exams (blood counts, C-reactive protein and erythrocyte sedimentation rate) and radiographs. If a specific signs are observed, second level investigations were considered (e.g. scintigraphy, magnetic resonance imaging, bone coltures).

In the meanwhile, empiric antibiotic therapy with teicoplanin and amikacin was begun. In case of positive coltures, a targeted antibiotic therapy was begun as soon as possible.

If CRP and microbial culture were negative, immediate soft tissue reconstruction was performed after washing and debridement, and empiric antibiotic therapy was continued for six weeks.

**Figure 1:**
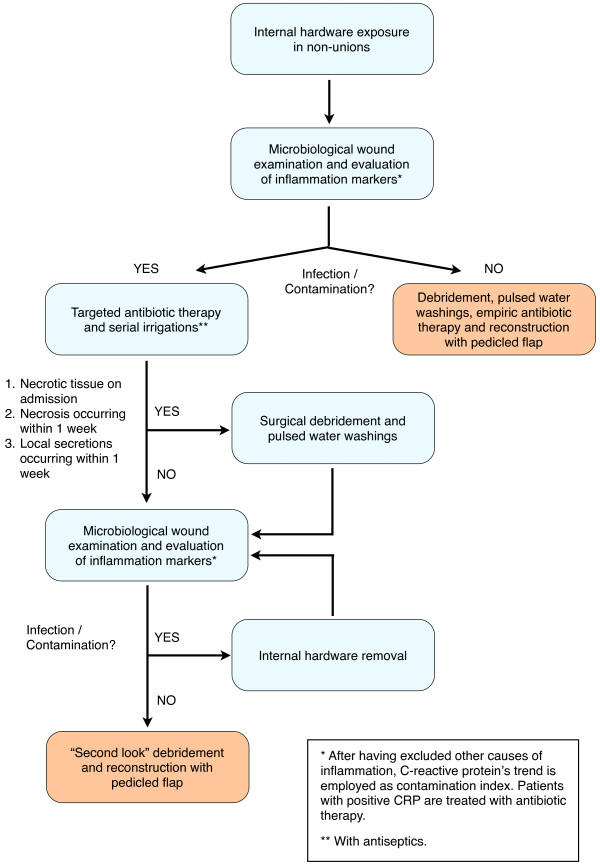
The immediate reconstructive strategy: operative diagram.

If CRP and/or microbial culture were positive, serial irrigations were performed daily with antiseptics and additional swabs were collected every 2 days until cultures became negative and CRP was lowering. Should it not occur within 12 days, the hardware would have been removed eventually. Otherwise, the soft tissue defect would have been reconstructed as soon as the CRP trend and the culture were negative (Figure 1).

**Figure 2:**
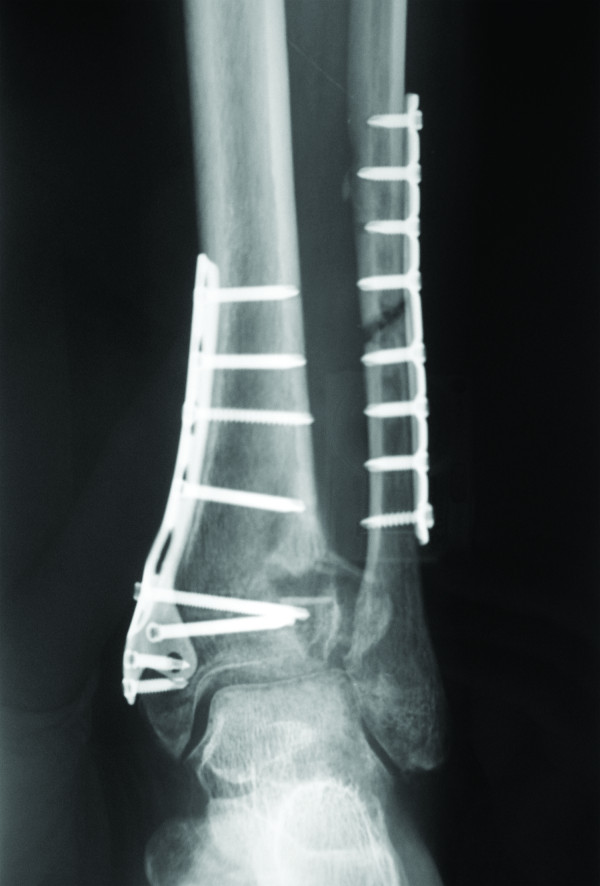
**The patient was referred to us with a wound measuring 0.5** × **0.5 mm with exposed internal hardware ( *left *).** After intra-operative debridement and excision of non-viable tissues, the wound measured approximatively 3 × 3 cm (*right*).

**Figure 3:**
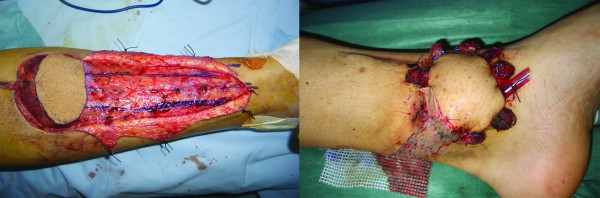
Pre-operative x-ray.

Whereas necrotic tissues were present on admission, if necrosis occurred within 1 week from the first administration of antibiotic therapy or if wound secretions were observed, surgical debridement was performed (Figures 2 and 3) and reconstruction with pedicled local or free flaps was considered when a layer of viable tissue was reached.

**Figure 4:**
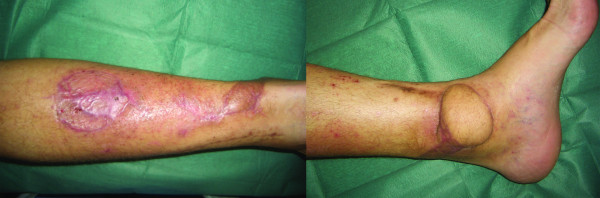
A sural fasciomiocutaneous flap is harvested and placed in the acceptor site.

Reconstruction was performed through pedicled flaps (sural-fasciocutaneous, sural fasciomiocutaneous or gastrocnemius flaps) or free flaps (anterolateral thigh free flap). The sural fasciomiocutaneous and gastrocnemius flaps are preferred by the authors due to their pliability that allow them to adapt to the shape and asperities of internal plates, maintaining a rich vascularization (Figure 4).

Before harvesting the flap, pulsed water washings with 5 liters of 5% povidone-iodine solution were carried out.

**Figure 5:**
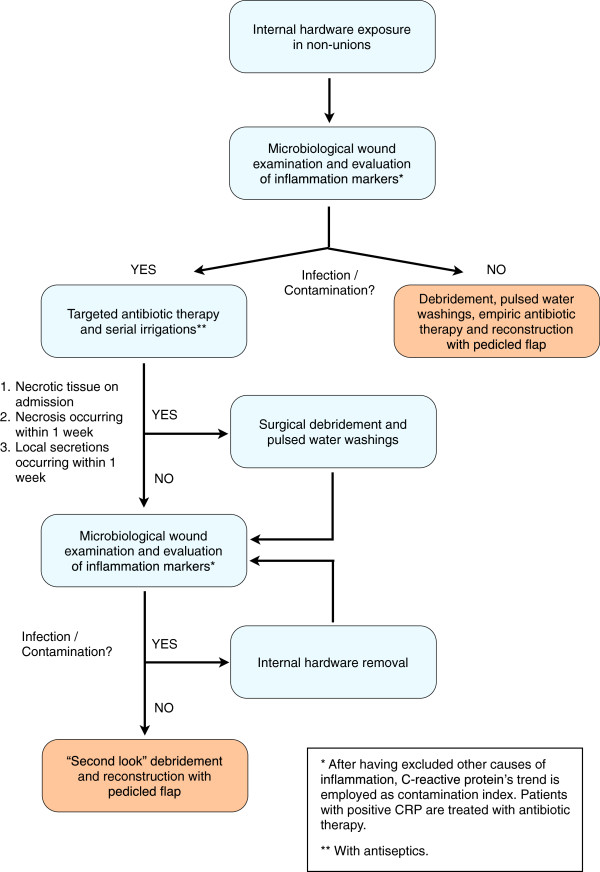
Post-operative result 3 months after surgery.

Patients were discharged 4 days after surgery, therefore they were followed as out-patients and visited every week for the first month. Minor complications (like wound dehiscence and superficial infection) were treated as out-patients (Figure 5). Major complication such as flap necrosis required prompt admission and debridement (Figure 1). Radiographs of the lower limb was obtained one year after surgery and signs of intramedullary, localized or diffuse osteomyelitis were searched in that occasion.

### Data collection

Demographic data were collected, along with hospital charts. Each patient was followed from the acceptance in our department until complete recovery was achieved, considering both fracture union and soft tissue healing without any dehiscence and/or infection.

All the patients affected by exposure of internal fixation devices after distal leg fracture, with infected either not-infected wounds, were included. Cases with inadequate follow-up (mainly due to distant place of residence), incomplete clinical documentation and orthopedic management different from ORIF were excluded.

The following anamnestic data were collected: localization of the internal hardware exposure, infections, reconstructive attempts of soft tissues, previous debridements were also recorded.

After soft tissue reconstruction with local pedicled or free flaps, complications (including necrosis, dehiscence and infection), substitution of internal hardware with external fixation and further surgeries were considered.

The variables concerning patient’s treatment were therefore divided in “potentially predictive variables” and “outcome variables” (Additional file 1: Table S1).

## Results

From July 2004 to March 2010, 13 patients underwent soft tissue reconstruction according to IRS due to internal hardware exposure and various-degree soft tissue damage. At the presentation c/o our departments of tissues infection was detected in 10 cases (76.9%) before surgery (mainly Enterococcus spp and Staphilococcus spp) and the wounds had an average measure of 38.2 ± 55.6 cm^2^.

The mean age at presentation was 46.4 years (ranging from 24 to 71) and the wound was present from an average of 5 months (9.89 months in cases affected by infection and 6.27 in patients without infection) In all cases the internal hardware was made by stainless steel.

In six patients the lesion was localized on the left lower limb, in seven patients on the right. In 15.4% of cases the wound lay at the lateral malleolus, in 23.1% on the medial malleolus and in eight cases (61.5%) along the distal third of tibia.

In all cases incomplete ossification at presentation was reported, in one case a soft tissue reconstruction was already attempted by local flaps.

In two cases, an eschar on more than 60% of the wound was observed.

Among infected patients, debridement of necrotic tissues was performed in five patients in order to achieve a sterile ground for the further soft tissue reconstruction. At the orthopedic follow-up, no signs of osteomyelitis have been reported (Additional file 2: Table S2 and Additional file 3: Table S3).

The following flaps were raised: sural fasciomiocutaneous in four cases, sural fasciocutaneous in three cases, three medial gastrocnemius muscle flaps, one soleus muscle flap, one perforator flap and one free anterolateral thigh flap. The mean time from internal hardware placement and soft tissue reconstruction was 13 months. After raising the flap, complications occurred in eight cases (61.5%). All of these cases were pre-operatively affected by soft tissues infection. No complications were observed in the other patients.

The observed complications include fistulization at flap‘s margin (five cases), necrosis (two cases) and dehiscence (one case). In those cases affected by fistulization (five patients), the coltures revealed the same of the infective agents observed before surgery.

In three cases the cutaneous dehiscence healed only after substitution of the internal hardware with an external fixator.

In two cases (15.4%), where the flap was affected by margin dehiscence and wound infection, the surgeon opted to perform debridement of necrotic and purulent tissues, 25 and 17 days after harvesting the flap.

In one case complete necrosis of the flap was observed six days after surgery (Additional file 4: Table S4 and Additional file 5: Table S5).

Assuming that no patients became infected after surgery, Mc Nemar test for paired samples showed no significative improvements in infection rate from the pre-operative period to the post-operative time (p = 0.125). Nevertheless a documented pre-operative infection is significantly associated with overall complications (p = 0.002), consisting in a predictive value of 0.89, while it is not associated with the necessity of further surgeries (p = 0.057), although this relationship is plausible.

When reconstruction was performed at more than six months from internal hardware application, higher rates of complications are observed (p = 0.028).

Apparently, wound size neither surgical debridement (performed in a previous surgery) have associations with the outcome variables, as much as the so-called “potentially predictive variables”. Surgical technique and complications have no statistically valuable nexus.

Full flap necrosis was observed in case two which was treated with a perforator flap. After flap failure, extensive debridement was performed and, in a further surgical time, a sural fasciocutaneous flap was harvested without hardware removal. The patient healed with no complications. Fracture fixation resulted stable in all cases and removal of internal hardware was generally unnecessary. In three cases internal hardware was removed for the application of external fixators in order to achieve complete healing of the wound where persistent wound infection and/or dehiscence was observed.

Among our case series, 10 patients came to our attention with exposed bone and infected wounds, belonging to stage 2 or superficial osteomyelitis according to Cierny-Mader classification [20]. No signs of intramedullary, localized or diffuse osteomyelitis (stages 1, 3 and 4) have been reported. After the treatment with IRS, no signs of osteomyelitis were observed.

## Discussion

The role of the reconstructive surgeon is essential in the management of high energy distal tibia fractures [21,22]. In fact even the recent minimally invasive internal fixation with locked plate systems still shows several complications concerning soft tissues. Ronga reports wound problems in 42.9% of patients treated with locked plating for distal tibial fractures [23]. Analogously, Namazi observed 23.5% of patients suffering of Locked Compression Plate exposure, who required soft tissue reconstruction in 37.5% [24]. Considered the high rates of internal hardware exposure requiring soft tissue reconstruction, an effective strategy is needed in treating this challenging problem. The main risks of internal hardware exposure are infections, osteomyelitis and non-unions. Therefore, goals of this strategy should consist first of all in the prevention of complications, at the same shortening hospitalization and allowing early mobilization.

According to the current indications concerning soft tissue reconstruction, internal hardware preservation in the lower limb may be attempted if exposure lasts less than two weeks and wound coltures are negative [17]. In the other cases, removal of the hardware is suggested, regardless of reconstruction which is not necessarily indicated.

In our case series, patients were referred to us five months after wound dehiscence on average and internal hardware exposure. None of these came to our attention within two weeks from hardware exposure. According to literature, this condition could have been treated by two different approaches.

First, after targeted antibiotic therapy and serial irrigations, debridement, removal of internal hardware and immediate soft tissue reconstruction could be performed (with/without placement of external fixators). Alternatively reconstruction could be delayed in order to treat the underlying infection with the proper targeted antibiotic therapy [17].

On the other hand, delayed reconstruction increases parallel the risk of nosocomial wound infections. For this reason the first approach could be preferred.

Nevertheless in literature no clear evidences have been reported about the advantages of removing the internal hardware before soft tissue reconstruction concerning complications. On the contrary some authors claim the possibility of immediate reconstruction [18,25].

In our case series, the most frequent complication was wound dehiscence and infection. Accounting that infection was present pre-operatively in 10 patients (76.9%), while after surgery signs of infection (wound dehiscence) were demonstrated in five cases (38.5%), a reduction of 38.4% was observed. Among these five cases, three patients required removal of internal hardware to eradicate infection and achieve full recovery. In one case the flap failed (a perforator flap) for inconsistent vascular supply and an ulterior surgery was needed to obtain definitive reconstruction with a sural fasciocutaneous flap, while in another case a surgical debridement of the flap was performed.

Overall, further surgeries after immediate reconstruction were needed in five cases, thus saving operations in eight patients (61.5%) who classically would have undergone a separate reconstructive surgery. Our protocol reduced hospitalization and allowed early mobilization. Moreover the affected area could immediately benefit from the new vascularization brought by the pedicled flap, especially for the carriage of antibiotics.

Although pre-operative infection was significantly associated with post-surgical complications (p = 0.014), the IRS turned out to be effective, accounting flap failure in one case.

## Conclusions

Internal hardware exposure in the lower limb is a common problem which involves up to 42.9% of patients with locked plating devices [23]. This issue is classically treated by hardware removal and could require delayed reconstruction with pedicled or free flaps in 37.5% of cases [24].

In our case series we performed immediate soft tissue reconstruction on exposed hardware after serial irrigations and targeted antibiotic therapy, achieving full recovery in 53.8% of cases after one surgery, therefore reducing hospitalization and allowing early mobilization (saving operations in 8 patients). However controlled trials are needed to confirm our encouraging results.

## Description

Case series of patients affected by internal fixator exposure treated with Immediate Reconstructive Strategy: retrospective analysis and outcomes.

## Abbreviations

IRS: Immediate Reconstructive Strategy.

## Competing interests

The authors declare that they have no competing interests.

## Authors’ contributions

DMM and VL have made substantial contributions to conception and design of the study. GR gave his substantial contribution in data collection, analysis and in manuscript writing. PL gave substantial contribution in data interpretation and manuscript revising. PG and MA have been involved in manuscript revision and data analysis. All authors read and approved the final manuscript. We did not receive in the past five years reimbursements, fees, funding, or salary from any organization that may in any way gain or lose financially from the publication of this manuscript, either now and in the future.We have no stocks or shares in any organization that may in any way gain or lose financially from the publication of this manuscript, either now and in the future. We are not applying any patent relating to the content of the manuscript and we received no reimbursements, fees, funding, or salary from any organization. We have no other financial competing interests.We have no non-financial competing interests (political, personal, religious, ideological, academic, intellectual, commercial or any other) to declare in relation to this manuscript.

## Supplementary Material

Additional file 1 **Table S1.** Considered variables. Click here for file

Additional file 2 **Table S2.** Patients not affected by wound infection [20]. Click here for file

Additional file 3 **Table S3.** Patients affected by wound infection. Click here for file

Additional file 4 **Table S4.** Patients not affected by wound infection - reconstruction and complications. Click here for file

Additional file 5 **Table S5.** Patients affected by wound infection - reconstruction and complications. Click here for file
