# Supplementary material for: First results with the immediate reconstructive strategy for internal hardware exposure in non-united fractures of the distal third of the leg: case series and literature review
Source: J Orthop Surg Res. 2012 Aug 28;7:30. doi: 10.1186/1749-799X-7-30 (PMC3489621; doi:10.1186/1749-799X-7-30)
Supplement: Additional file 1 — Table S1. Considered variables. [file 1749-799X-7-30-S1.doc]

*Table 1* - Considered variables

| **Potentially predictive variables** | **Outcome variables** |
| --- | --- |
| Age > 50 years | Overall complications |
| Eschar at presentation on more than 60% of the wound | Post-operative infection |
| Surgery after more than 6 months from internal hardware application | Further surgeries |
| Pre-operative negative-pressure dressings | Complete flap necrosis |
| Surgical debridement before reconstruction in a different surgical time | Partial flap necrosis |
| Documented infection pre-operatively | Overal flap necrosis |
| Wound measuring >= 30 cm2 |  |
