# Supplementary material for: First results with the immediate reconstructive strategy for internal hardware exposure in non-united fractures of the distal third of the leg: case series and literature review
Source: J Orthop Surg Res. 2012 Aug 28;7:30. doi: 10.1186/1749-799X-7-30 (PMC3489621; doi:10.1186/1749-799X-7-30)
Supplement: Additional file 2 — Table S2. Patients not affected by wound infection [20]. [file 1749-799X-7-30-S2.doc]

Table 2: Patients not affected by wound infection

| N | Sex | Age | Wound localization | Fracture type | Lower limb | Wound area (cm^2^) | Eschar | Wound infection etiology | Cierny- Mader stage [1] | Surgical debridement | Internal hardware |
| --- | --- | --- | --- | --- | --- | --- | --- | --- | --- | --- | --- |
| 1 | M | 40 | Medial malleolus | Malleolar | Left | 9 | - | None | - | - | Plate and screws |
| 2 | M | 56 | Anterior | Tibial pilon | Right | 3 | - | None | - | - | Plate and screws |
| 3 | F | 40 | Anterior | Tibial pilon | Left | 80 | Yes | None | - | - | Plate and screws |

Bibliography

1. Cierny G, Mader JT, and P. H., A clinical staging system of adult osteomyelitis*.* Contemp Orthop, 1985. 10: p. 17–37
