# Supplementary material for: First results with the immediate reconstructive strategy for internal hardware exposure in non-united fractures of the distal third of the leg: case series and literature review
Source: J Orthop Surg Res. 2012 Aug 28;7:30. doi: 10.1186/1749-799X-7-30 (PMC3489621; doi:10.1186/1749-799X-7-30)
Supplement: Additional file 3 — Table S3. Patients affected by wound infection. [file 1749-799X-7-30-S3.doc]

Table 3: Patients affected by wound infection

| N | Sex | Age | Wound localization | Fracture type | Lower limb | Wound area (cm2) | Eschar | Wound infection etiology | Cierny- Mader stage ^1^ | Surgical debridement | Internal hardware |
| --- | --- | --- | --- | --- | --- | --- | --- | --- | --- | --- | --- |
| 1 | F | 71 | Medial malleolus | Malleolar | Left | 66 | Yes | Enterococcus cloacae, Escherichia coli | 2A | Yes | Screws |
| 2 | F | 46 | Anterior | Tibial pilon | Right | 200 | - | Staphilococcus chromogenes, Alcaligenes spp, Pseudomonas aeruginosa | 2B-l | - | Plate and screws |
| 3 | F | 61 | Medial malleolus | Malleolar | Left | 0.3 | - | Gram positive cocci | 2A | - | Plate and screws |
| 4 | F | 70 | Medial malleolus | Malleolar | Right | 50 | - | Enterococcus cloacae | 2A | - | Plate and screws |
| 5 | M | 50 | Anterior | Tibial pilon | Left | 42 | - | Staphilococcus epidermidis | 2B-l | Yes | Plate and screws |
| 6 | M | 40 | Anterior | Tibial pilon | Right | 4 | - | Enterobacter cloacae, Klebsiella pneumoniae | 2B-l | - | Plate and screws |
| 7 | M | 28 | Anterior | Tibial pilon | Right | 30 | - | Enterococcus spp, Vibrio alginolyticus and Pseudomonas stutzeii | 2A | Yes | Plate and screws |
| 8 | M | 39 | Lateral malleolus | Malleolar | Left | 4 | - | Gram positive cocci | 2A | - | Plate and screws |
| 9 | M | 31 | Anterior | Tibial pilon | Right | 4 | - | Staphilococcus aureus | 2A | Yes | Plate and screws |
| 10 | M | 24 | Anterior | Tibial pilon | Right | 5 | Yes | Bacillus spp | 2A | Yes | Plate and screws |

Bibliography

1. Cierny G, Mader JT, H. P (1985) A clinical staging system of adult osteomyelitis Contemp Orthop: 10; 17–37
