# Supplementary material for: First results with the immediate reconstructive strategy for internal hardware exposure in non-united fractures of the distal third of the leg: case series and literature review
Source: J Orthop Surg Res. 2012 Aug 28;7:30. doi: 10.1186/1749-799X-7-30 (PMC3489621; doi:10.1186/1749-799X-7-30)
Supplement: Additional file 4 — Table S4. Patients not affected by wound infection - reconstruction and complications. [file 1749-799X-7-30-S4.doc]

Table 2: Patients not affected by wound infection - reconstruction and complications.

| N | Performed flap | Post-operative complications | Post-operative infection | Infective agent | Further surgeries | Overall surgeries required for complete wound healing |
| --- | --- | --- | --- | --- | --- | --- |
| 1 | Sural fasciomiocutaneous | - | - | - | - | 1 |
| 2 | Medial gastrocnemius | - | - | - | - | 1 |
| 3 | Free anterolateral thigh | - | - | - | - | 1 |
