# Supplementary material for: First results with the immediate reconstructive strategy for internal hardware exposure in non-united fractures of the distal third of the leg: case series and literature review
Source: J Orthop Surg Res. 2012 Aug 28;7:30. doi: 10.1186/1749-799X-7-30 (PMC3489621; doi:10.1186/1749-799X-7-30)
Supplement: Additional file 5 — Table S5. Patients affected by wound infection - reconstruction and complications. [file 1749-799X-7-30-S5.doc]

Table 2: Patients affected by wound infection - reconstruction and complications.

| N | Performed flap | Post-operative complications | Post-operative infection | Infective agent | Further surgeries | Overall surgeries required for complete wound healing |
| --- | --- | --- | --- | --- | --- | --- |
| 1 | Sural fasciomiocutaneous | Infection and dehiscence | Yes | E. Coli | Debridement | 3 |
| 2 | Perforator | Full flap necrosis | - | - | Flap removal and debridement. Sural fasciocutaneous flap in a further surgery | 3 |
| 3 | Sural fasciomiocutaneous | Infection and dehiscence | Yes | Gram + cocci | - | 1 |
| 4 | Sural fasciomiocutaneous | Dehiscence, partial necrosis | - | - | - | 1 |
| 5 | Medial gastrocnemius | - | - | - | - | 2 |
| 6 | Soleus | Infection and dehiscence | Yes | Enterobacter Cloacae | Internal hardware removal and application of external fixators | 1 |
| 7 | Medial gastrocnemius | Infection and dehiscence | Yes | Vibrio alginolyticus | Debridement, internal hardware removal and application of external fixators | 3 |
| 8 | Sural fasciocutaneous | Infection and dehiscence | Yes | Gram positive cocci | - | 1 |
| 9 | Sural fasciocutaneous | - | - | - | - | 2 |
| 10 | Sural fasciocutaneous | Dehiscence | - | - | Internal hardware removal and application of external fixators | 2 |
